# Epstein-Barr virus–associated hemophagocytic syndrome mimicking severe sepsis

**DOI:** 10.4103/0974-2700.43198

**Published:** 2008

**Authors:** Talya Spivack, Rashmi Chawla, Paul E Marik

**Affiliations:** Department of Internal Medicine; 1 Division of Pulmonary and Critical Care Medicine, Thomas Jefferson University, Philadelphia, PA, USA

**Keywords:** Bile ductopenia, Epstein-Barr virus, erythrophagocytic syndrome, hemophagocytic lymphohistiocytosis, hemophagocytic syndrome, multiorgan failure, sarcoid, sepsis, systemic inflammation

## Abstract

Severe sepsis is amongst the most common reasons for admission to the intensive care unit (ICU) throughout the world and is a common cause of death. The diagnosis of sepsis is usually straightforward, being based on a constellation of clinical and laboratory features. Noninfectious disorders, including pancreatitis, drug reactions, and autoimmune disorders, may cause a systemic inflammatory response that mimics sepsis. We present the case of a 32-year-old male with Epstein-Barr virus-associated hemophagocytic syndrome who presented to the ICU with features of severe sepsis which progressed to multisystem organ failure and death despite aggressive supportive measures.

## INTRODUCTION

Sepsis is amongst the most common reasons for admission to intensive care units throughout the world and the leading cause of death in noncoronary ICUs.[1,2] The diagnoses of sepsis is usually straightforward; patients present variably with tachycardia, tachypnea, fever (hypothermia), leukocytosis (or leukopenia and bandemia), together with a hyperdynamic circulation and a presumed source of infection.[3] These manifestations are largely due to cytokine release triggered by bacterial products. Noninfectious disorders, including pancreatitis, drug reactions, and autoimmune disorders, may cause a systemic inflammatory response that mimics sepsis. We report a patient with Epstein-Barr virus (EBV)–associated hemophagocytic syndrome (HPS) who presented with the features of severe sepsis. 

## CASE REPORT

A 32-year-old male was admitted to our hospital with persistent fever, body aches, and rhinorrhea of 2 weeks' duration. He had recently completed a course of antibiotics for presumed acute sinusitis. He denied having cough, chest pain, abdominal pain, dysuria, or headaches. He had a history of worsening jaundice and pruritus over the previous 4 months. He gave a history of heavy alcohol abuse in the past. He worked as a correctional officer and enjoyed hunting wild animals. A liver biopsy performed at another hospital 2 months earlier for presumed alcoholic hepatitis had revealed ‘scattered noncaseating granulomas within the parenchyma and portal tracts’; culture and stain for mycobacterium had been negative. These features were considered to be consistent with hepatic sarcoidosis and treatment with oral prednisone at a dose of 20 mg daily had been commenced.

On presentation to our hospital, he was markedly jaundiced, febrile (39.4°C), and hypotensive. He was alert and orientated. Physical examination revealed hepatosplenomegaly and small and shotty right axillary lymphadenopathy. He was pancytopenic; hemoglobin (Hb) was 8.7 g/dl, with 1.6% reticulocytes; white blood cell count (WBC) was 1100/μL with 66% neutrophils and 18% bands and a platelets count of 104 000/μL. Serum chemistries were normal. Total bilirubin was 33 mg/dl (normal: 0.2–1.2 mg/dl), direct bilirubin 14 mg/dl (normal: 0.0–0.4 mg/dl), alanine aminotransferase (ALT) 135 IU/l (normal: 1–45 IU/l), aspartate aminotransferase (AST) 25 IU/l (normal: 7–42 IU/l), alkaline phosphatase (ALP) 1431 IU/l (normal: 25–120 IU/l), lactate dehydrogenase 411 IU/l (normal: 100–200 IU/l), ceruloplasmin 27.2 mg/dl (normal: 22–58 mg/dl), fibrinogen 138 mg/dl (normal: 201–422 mg/dl), D-dimer 0.68 ug/ml (normal: < 0.68 ug/ml), triglycerides 161 mg/dl, and ferritin 5208 ng/ml (normal: 15–280 ng/ml). Immunoglobulin electrophoresis was normal. Portable chest radiograph was reported to be normal. Although there was no obvious source of infection, he was treated with vancomycin and piperacillin/tazobactam for presumed severe sepsis. His hypotension resolved with fluid resuscitation (2 l of 0.9% saline). Blood and urine cultures were negative. HIV; cytomegalovirus; parvovirus; hepatitis A, B, and C; coccidioides; toxoplasmosis; and brucella serologies were negative. Epstein-Barr virus (EBV) IgG was positive, consistent with previous exposure. Autoimmune workup, including ANA, ANCA, rheumatoid factor, and Coomb's test, were negative. Computerized tomographic scans of his head, chest, and abdomen showed extensive pansinusitis, innumerable scattered bilateral lung nodules measuring up to 1 cm in diameter, mild right axillary lymphadenopathy, and hepatosplenomegaly. A bone marrow biopsy and aspirate revealed a cellular marrow containing all normal hematopoietic elements with left-shifted myelopoiesis and no evidence of malignancy or granulomatous disease; stain for acid fast bacilli was negative.

**Figure 1:**
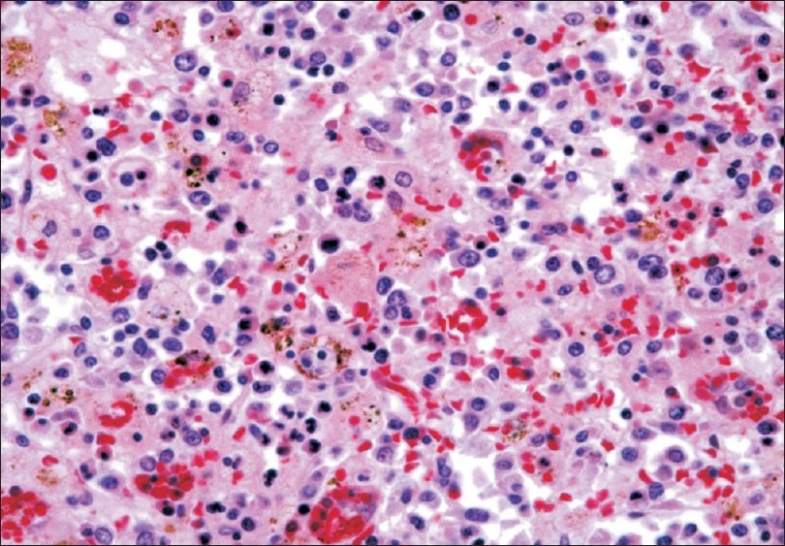
Hematoxylin-eosin stain of liver sample obtained at autopsy showing numerous phagocytic cells with engulfed hematopoietic elements

The patient's hospital course was complicated by persistent fever (39.2°C), with progressive pancytopenia (Hb 7.4 g/dl, WBC 600/μL, platelets 14/μL) and recurrent episodes of fluid-responsive hypotension. A 4-drug antituberculous regimen for possible miliary tuberculosis was started. As there was no clinical improvement with treatment over the next few days, a repeat liver biopsy was performed; this showed ductopenia with no granulomas. At this point, we considered the possibility of the HPS and the patient was treated with high-dose methylprednisolone (1 g daily) and polyclonal intravenous immunoglobulin (1 g/kg).[4] However, the patient developed volume-refractory hypotension requiring escalating doses of vasopressor agents and mechanical ventilation for progressive hypoxemia. His condition rapidly deteriorated and he suffered a cardiac arrest from which he could not be resuscitated. The family agreed to an autopsy An EBV PCR sent prior to his death demonstrated 692,000 copies/ml of the virus; PCR for CMV was negative. Autopsy confirmed the diagnosis of HPS; there was involvement of liver, spleen, bone marrow, lymph nodes, maxillary sinuses, and lung parenchyma, which demonstrated infiltration with hypertrophic macrophages and erythrophagocytosis [Figure 1]. Electron microscopy demonstrated hypertrophic macrophages ‘stuffed with phagocytic debris.’ Molecular studies on postmortem bone marrow, liver, spleen, lung, and liver specimens were positive for EBV. Postmortem cultures for all other viral, fungal, and acid-fast organisms were negative. Gene mutation analysis for X-linked lymphoproliferative disease (BIRC4 and SH2DD1A genes) was negative. Review of the initial liver biopsy was consistent with lymphocytic hepatitis (primary EBV hepatitis).

## DISCUSSION

HPS, also called hemophagocytic lymphohistiocytosis (HLH), is a well-defined clinical disease. It was first described by Scott and Robb-Smith in 1939.[5] HPS is a condition in which T cells, natural killer cells, and macrophages are aberrantly activated, resulting in hypercytokinemia that causes cellular damage leading to mutiorgan failure.[6,7] Markedly elevated levels of tumor necrosis factor-alpha (TNF-alpha), soluble interleukin-2 receptor (sIL-2R), interferon-gamma (IFN-gamma), IL-6, IL-10, and IL-18 have been reported in the active phase of the disease.[6,8,9] HPS is characterized by prolonged fever; hepatosplenomegaly; cytopenia; coagulopathy; hypertriglyceridemia; and erythrophagocytosis in the bone marrow, liver, spleen, or lymph nodes.[6,7,10] Two forms of HPS have been defined; the primary or familial and the sporadic or reactive type [Table 1].[6,10] The reactive type of HPS may be associated with infections, autoimmune disorders, or malignancies. Among the infections associated with HPS, Epstein-Barr virus (EBV) is the most common.[6,10]

**Table 1:** Classification and underlying conditions associated with the hemophagocytic syndrome

Genetic HPS
Familial HPS (Farquhar's disease)
Known genetic defects (perforin, munc 13-4, syntaxin 11
Unknown gene defects
Immune deficiency syndromes
Chediak-Higashi syndrome
Griscelli syndrome
X-linked lymphoproliferative syndrome
Acquired HPS
Exogenous agents (toxins)
Infection-associated hemophagocytic syndrome (Herpes virus, adenovirus, parvovirus, influenza, hepatitis viruses)
Macrophage activation syndrome (associated with autoimmune diseases)
Malignant diseases
HPS: Hemophagocytic syndrome

Diagnosis of the HPS syndrome is based upon the clinical, laboratory, and histopathological findings. Diagnostic guidelines were proposed by the Histiocytic Society in 1991 and updated in 2004[11,12] [Table 2]. Our patient had six of the eight diagnostic criteria. HPS should be considered in the differential diagnosis in patients presenting with prolonged fever, pancytopenia, and a sepsis-like picture which is unresponsive to antibiotics. Since therapy can be life saving and some of the clinical criteria occur late in the disease, it is not necessary to wait for fulfillment of all criteria before initiating therapy. As in our case, hemophagocytosis may be absent on initial bone marrow biopsy and it may have to be repeated.[7] The presence of a high EBV DNA load in the blood facilitates the diagnosis of EBV-HPS.[13] CD8-positive T/NK cells with positive immunohistochemical staining for EBV-LMP1 and EBV-EBNA2 has also been demonstrated.[14] Bile ductopenia was noted on our patient's second liver biopsy. This finding has been reported previously in patients with EBV-associated HPS and it may be an EBV-associated phenomenon.[15,16]

**Table 2:** Diagnostic criteria of the hemophagocytic syndrome (HPS)[12,18]

The diagnosis of HPS is established of fulfillment of one of the following criteria
A molecular diagnosis consistent with hemophagocytic syndrome (e.g., PRF mutations, SAP mutations, MUNC13-4 mutations
Five out of eight of the following
Fever
Splenomegaly
Cytopenia (two cell lines)
Hypertriglyceridemia (> 265 mg/dl) and/or hypofibrinogenemia (< 150 mg/dl)
Hemophagocytosis in the bone marrow, spleen, or lymph nodes, without evidence of malignancy
Low or absent natural killer cell cytotoxicity
Hyperferritinemia (> 500 ng/ml)
Elevated soluble CD 25 (interleukin-2R alpha chain > 2400 IU/ml)

The epidemiology of EBV-HPS is not well understood. Although the disease occurs most commonly in children and adolescents living in Japan, Taiwan, and other Asian counties, it has also been reported to occur sporadically in Western countries.[6,13] A highly pathogenic stain of EBV may account for the higher incidence in Asian countries. In adults EBV-HPS is usually a fatal disease in which patients die from progressive multiorgan failure.[10,13–15] Primary EBV infection occurring in early childhood is usually asymptomatic. Primary infection in adolescents and young adults may cause infectious mononucleosis, in which EBV infects B cells, with polyclonal B cell expansion accompanied by antigen-driven oligoclonal on monoclonal proliferation of CD8-positive cytotoxic T cells. In EBV-HPS, EBV infects CD8-positive T cells, leading to polyclonal or monoclonal proliferation of EBV-containing T cells. Although the pathogenesis of EBV-HPS has not yet been fully elucidated, inflammatory cytokines, especially TNF-alpha, produced by EBV-infected T cells or macrophages, are thought to be responsible for the HPS. TNF-alpha may be particularly important in the pathogenesis of HPS as it is a potent activator of macrophages. Lay and colleagues have demonstrated that late membrane protein-1 (LMP1), a gene product of EBV-infected T cells, upregulates the TNF-alpha gene.[17]

The HPS is a highly fatal disease if untreated. The immediate aim of therapy is suppression of the increased inflammatory response (cytokine storm) and control of T cell proliferation using immunosuppressive and cytotoxic drugs. Treatment differs in children and adults and depends on the underlying disease, the presence of a trigger, and the severity of symptoms. EBV-specific therapy is ineffective in treating EBV-HPS.[10] Chemotherapy using dexamethasone, cyclosporin A, and etoposide was adopted by the Histiocyte Society in 1994 (updated 2004) and is used for severe (familial and EBV-HPS) cases.[12,18] Cyclosporine A prevents T cell activation. Etoposide inhibits EBV nucleic acid synthesis in EBV-infected cells and has cytotoxic activity in monocytic and histiocytic diseases. EBV-associated HPS was previously thought to be fatal in most cases; however, the introduction of an etoposide-based regimen has greatly improved survival times.[6,19]

## CONCLUSION

HPS is a rare disorder resulting from the uncontrolled production of inflammatory cytokines and a clinical presentation that mimics sepsis. Delayed diagnosis usually results in a fatal outcome. HPS usually runs an aggressive course and requires a specific therapeutic regimen for cure. Clinicians need to understand the pathophysiology of this disease to facilitate its early recognition and treatment.
